# Dissecting the Prefrontal Network With Pathway-Selective Manipulation in the Macaque Brain—A Review

**DOI:** 10.3389/fnins.2022.917407

**Published:** 2022-05-23

**Authors:** Mineki Oguchi, Masamichi Sakagami

**Affiliations:** Brain Science Institute, Tamagawa University, Tokyo, Japan

**Keywords:** prefrontal network, pathway selectivity, brain manipulation, non-human primate, macaque monkey, chemogenetics, optogenetics

## Abstract

Macaque monkeys are prime animal models for studying the neural mechanisms of decision-making because of their close kinship with humans. Manipulation of neural activity during decision-making tasks is essential for approaching the causal relationship between the brain and its functions. Conventional manipulation methods used in macaque studies are coarse-grained, and have worked indiscriminately on mutually intertwined neural pathways. To systematically dissect neural circuits responsible for a variety of functions, it is essential to analyze changes in behavior and neural activity through interventions in specific neural pathways. In recent years, an increasing number of studies have applied optogenetics and chemogenetics to achieve fine-grained pathway-selective manipulation in the macaque brain. Here, we review the developments in macaque studies involving pathway-selective operations, with a particular focus on applications to the prefrontal network. Pathway selectivity can be achieved using single viral vector transduction combined with local light stimulation or ligand administration directly into the brain or double-viral vector transduction combined with systemic drug administration. We discuss the advantages and disadvantages of these methods. We also highlight recent technological developments in viral vectors that can effectively infect the macaque brain, as well as the development of methods to deliver photostimulation or ligand drugs to a wide area to effectively manipulate behavior. The development and dissemination of such pathway-selective manipulations of macaque prefrontal networks will enable us to efficiently dissect the neural mechanisms of decision-making and innovate novel treatments for decision-related psychiatric disorders.

## Introduction

The prefrontal cortex (PFC) is located in the anterior part of the brain, and subdivided into heterogeneous and cytoarchitecturally diverse areas, such as the ventral prefrontal cortex (VLPFC), dorsolateral prefrontal cortex (DLPFC), orbitofrontal cortex (OFC), medial prefrontal cortex (MPFC), frontal eye field (FEF), etc. These prefrontal regions have complex connections with one another, and their posterior portions, in particular, have long-range connections with the more posterior cortical and subcortical regions (Haber et al., 2022). Through these complex neural circuits, the PFC builds adaptive decisions by filtering and integrating relevant sensory, memory, and internal information, and outputs them into motor plans (Miller, 2000; Miller and Cohen, 2001; Koechlin, 2014, 2016; Tanaka et al., 2015). To dissociate the functions of these prefrontal networks, a fine-grained method that selectively controls individual neural pathways is required. It is especially important to manipulate neural circuits using non-human primates, which have a high degree of kinship with humans, to better understand the prefrontal mechanisms of human decision-making. The macaque monkey, the largest primate for which neurophysiological interventions are available, has a well-developed prefrontal cortex that is highly homologous to the human brain, and can be trained to perform complex behavioral tasks with highly sophisticated hand and eye movements, making it an excellent animal model.

Conventional interventions in the macaque PFC, such as aspiration and transection, electrical microstimulation, local cooling, and microinjection of agonists and antagonists, activate or suppress the activity of neuronal populations near intervention sites, in specific prefrontal areas. However, these methods affect various neurons that are part of multiple neural circuits that pass through an area. Therefore, even if a reasonable change is observed in a behavioral task related to a certain function when brain activity is manipulated with these interventions, it is not possible to determine which neural circuit in the prefrontal network is responsible for that function. In the last decade, neural manipulation using transgenic techniques, such as optogenetics and chemogenetics, has become widespread in non-human primates (for review, Galvan et al., 2018; Tremblay et al., 2020; Klink et al., 2021). Some of these studies have achieved cell type-specific manipulation using specific promoters, pathway-selective manipulation by light stimulation or ligand administration to axon terminals of neurons expressing artificial receptors, or by systemic ligand administration with double-viral vector transduction. Furthermore, technological developments to facilitate such fine-grained neural manipulations have also been made from various directions, including the improvement of highly effective viruses for the brains of non-human primates, development of devices for effective infection and stimulation across large areas of the brain, and large-scale or multichannel neural recordings simultaneously with manipulations.

The prefrontal cortex is an intermediate area where highly abstract processing takes place to convert concrete sensory information into concrete motor information according to relevant contexts (Wallis et al., 2001; Pan et al., 2008, 2014; Antzoulatos and Miller, 2014; Wutz et al., 2018). Thus, it is difficult to map properly articulated functions to specific pathways in the prefrontal network. This would require, for instance, a systematically designed experimental paradigm that manipulates multiple prefrontal pathways for the same task designed to factorize prefrontal function. Recently, with the technological developments described above, studies that embody such research directions have begun to emerge, with pathway-selective manipulations in the macaque PFC.

In this review, we first provide an overview of traditional methods of non-pathway-selective manipulation of the macaque PFC and discuss their limitations. Next, we outline recent studies that have applied pathway-selective manipulation in non-human primates, and introduce attempts to develop new technologies to facilitate such interventions. Pathway-selective manipulations used in non-human primates include the blockade of signal transmission by tetanus neurotoxin using the “Tet-on” double-viral vector transduction, optogenetic manipulation by light stimulation of axon terminals or doubly transduced cell bodies, and chemogenetic manipulation by local administration of the ligand on axon terminals or systemic administration with double transduction. These pathway-selective methods will be reviewed, with a particular emphasis on studies using chemogenetics in the prefrontal network. We also show the development of viral vectors for the efficient expression of artificial receptors in non-human primates and devices for the efficient delivery of ligands and photostimuli. In the concluding sections, we briefly discuss the prospects of linking pathway-selective manipulation to the elucidation of pathology and novel therapies for psychiatric disorders and future challenges in understanding prefrontal functions in decision-making through pathway-selective manipulation.

## Traditional Approaches to Control Prefrontal Activity in the Macaque Brain

The logic of functional research using manipulation of brain activity is that if an intervention in a brain region causes a disturbance in a particular behavior, then that brain region is responsible for that behavior. Ablation studies on the prefrontal cortex of macaque monkeys have been conducted for several decades (Fuster, 2015). Recent studies have examined the differences in function between cortical regions, including those in the PFC, by systematically ablating each region and analyzing whether and how it affects task behavior (Mansouri et al., 2015, 2020). Although these studies provide interesting insights, the destruction of a brain site is irreversible, and may result in the formation of compensatory circuits, morphological changes at other brain sites, or long-lasting blood flow changes. In contrast, microinjection of muscimol, a gamma-amino butyric acid-A (GABA-A) agonist, reversibly suppressed neural activity at the injection site for several hours. Previous studies administered muscimol to the DLPFC during oculomotor response tasks and investigated the affected response fields and conditions (Sawaguchi and Iba, 2001; Kuwajima and Sawaguchi, 2007). Similarly, local cooling by pressing a metal cooling chamber against the cortical surface of the LPFC or inserting a cryoloop with coolant flowing into the principal sulcus has been used as a reversible manipulation technique (Chafee and Goldman-Rakic, 2000; Johnston et al., 2014, 2016; Koval et al., 2014; Chan et al., 2015). Electrical microstimulation of the PFC has also been used for a long time in macaque monkeys, and various studies have examined its effects on behavior by stimulating at a specific time in a behavioral task, taking advantage of its high temporal resolution compared to other methods (Moore and Fallah, 2001, 2004; Moore and Armstrong, 2003; Murphey and Maunsell, 2008; Schwedhelm et al., 2017). Although transcranial magnetic stimulation (TMS) is frequently used in human studies, it is primarily applied to macaque monkeys to investigate the neural mechanisms underlying TMS effects with electrophysiological recordings (Gu and Corneil, 2014; Honda et al., 2021).

Although the effects of these manipulations are local and non-pathway-selective, some studies have attempted to reveal neuronal circuit functions using electrophysiological recordings in other downstream areas or whole-brain functional magnetic resonance imaging (fMRI). For example, a previous study examined the interaction between the LPFC and parietal cortex of macaques during a delayed response task by performing single-unit recordings from one area during the local cooling of the other (Chafee and Goldman-Rakic, 2000). Similarly, another study examined the behavioral and neural effects of inactivation while applying local cooling to the DLPFC, and recorded spikes and local field potentials (LFPs) from the superior colliculus (Johnston et al., 2014; Chan et al., 2015).

In addition, in a recent study, the VLPFC was inactivated with muscimol injection, and neural activity was recorded from the inferior temporal cortex (IT) with a multicontact electrode array, revealing that the feedback signal from the VLPFC to the IT played a key role in the recognition of difficult (“late-solved”) objects (Kar and DiCarlo, 2021). This study also showed that IT activity during VLPFC inactivation resembled a feedforward neural network model. Several studies have combined electrical microstimulation with resting-state or task-related fMRI to examine the effects of microstimulation in other brain areas functionally connected to the stimulation site (Tolias et al., 2005; Ekstrom et al., 2008; Field et al., 2008; Moeller et al., 2008; Logothetis et al., 2010; Matsui et al., 2011, 2012; Klink et al., 2021). For instance, one study examined how microelectrical stimulation of the FEF during an eye movement task in an MRI scanner modulated top-down effects on the visual cortex (Premereur et al., 2013).

These studies provide important insights into the function of prefrontal networks. However, as some authors have admitted, these intervention methods cannot distinguish whether behavioral and neural changes are mediated through monosynaptic connections or multisynaptic connections through other cortical and subcortical regions. Furthermore, local ablation and electrical microstimulation affect not only neurons whose cell bodies are at the site of intervention, but also those whose fibers pass through that site. For example, behavioral changes found mainly in aspiration experiments on the macaque OFC may be due to inadvertent damage to the uncinate fascicles that pass through the OFC (Murray and Rudebeck, 2018). Indeed, Rudebeck and colleagues used an excitotoxin (ibotenic acid) that acts only on the cell body and not on the fibers to separately stimulate the OFC and VLPFC, with the latter being connected to the temporal lobe by the uncinate fascicles. They obtained results suggesting that some functions previously attributed to the OFC were actually carried out by the VLPFC (Rudebeck et al., 2017). These studies indicate that more fine-grained intervention methods that can work selectively on different pathways are required to investigate neural circuit function.

## Pathway-Selective Manipulation of the Macaque Prefrontal Network

Pathway-selective manipulation of neural activity has been made possible by the application of genetic engineering techniques. Because of the difficulty in efficiently producing transgenic macaques with germline modifications, genetic engineering of macaque monkeys has been performed primarily through the injection of viral vectors. In this section, we review pathway-selective manipulation in macaque monkeys using (1) Tet-on TeNT, (2) optogenetics, and (3) chemogenetics (Table 1).

**TABLE 1:** Summary of publications using pathway-selective manipulation methods in non-human primates.

Publication	Species	Method	Virus vector			Pathway
			
			Virus	Promotor	Transgene	
**Tet-on TeNT**						
Kinoshita et al., 2012	*Macaca fuscata*/ *mulatta*	Double transduction	AAV2 and HiRet	CMV	eTeNT	Propriospinal neurons
Tohyama et al., 2017	*Macaca fuscata*	Double transduction	AAV2/DJ and HiRet/ FuG-E/ NeuRet	CMV	eTeNT	Propriospinal neurons
Kinoshita et al., 2019	*Macaca fuscata*/ *mulatta*	Double transduction	AAV1 and HiRet	CMV	eTeNT	Superior colliculus to pulvinar
Ninomiya et al., 2020	*Macaca fuscata*	Double transduction	AAV-DJ and rAAV2.retro	CMV	eTeNT	Premotor cortex to MPFC
Vancraeyenest et al., 2020	*Macaca mulatta*	Double transduction	AAV2 and HiRet	CMV	eTeNT	The ventral tegmental area to nucleus accumbens
**Optogenetics**						
Inoue et al., 2015	*Macaca mulatta*	Single transduction	AAV2	CMV	ChR2	FEF to Superior colliculus
Galvan et al., 2016	*Macaca mulatta*	Single transduction	AAV5	CaMKIIα	ChR2/C1V1	Primary motor/Premotor cortices to thalamus
O’Shea et al., 2018	*Saimiri sciureus*	Double transduction	AAV2 and AAV8	Ef1α	ChR2	Premotor cortex to primary motor cortex
Nurminen et al., 2018	*Callithrix jacchus*	Cre-dependent mixture	AAV9	CaMKIIα	ArchT	Secondary visual cortex to primary visual cortex
Maeda et al., 2020	*Macaca mulatta*	Single transduction	AAV2	CMV	ChR2	Amygdala to substantia nigra pars reticulate
Amita et al., 2020	*Macaca mulatta*	Single transduction	AAV2	CMV	ChR2	Caudate tail to substantia nigra pars reticulate
**Chemogenetics**						
Oyama et al., 2021	*Macaca fuscata*/ *mulatta*	Single transduction	AAV1	hSyn	hM4Di	LPFC to thalamus/CdN
Oguchi et al., 2021b	*Macaca fuscata*	Double transduction	AAV5 and NeuRet	hSyn	hM4Di	LPFC to CdN

### Tet-On TeNT

The first method uses double-viral vector transduction to express neurotoxins (for a detailed review, see Isa, 2022). In this method, an anterograde vector that infects the cell body is injected at the departure area of the target pathway, and a retrograde vector that infects the axon terminals is injected at the destination area. The retrograde vector incorporates the gene sequence for enhanced tetanus toxin (eTeNT) downstream of the tetracycline response element (TRE). The anterograde vector incorporates the Tet-on sequence, which is a variant of the reverse tetracycline transactivator (rtTAV16). Doxycycline (Dox), a derivative of tetracycline, binds to rtTAV16 with oral administration, and induces gene expression downstream of TRE. eTeNT is then expressed only in doubly transduced neurons, inhibiting neurotransmitter release at axon terminals, thus blocking synaptic transmission in these neurons.

Tet-on TeNT has been applied to the spinal cord and subcortical and cortical regions of macaque monkeys (Kinoshita et al., 2012, 2019; Tohyama et al., 2017; Ninomiya et al., 2020; Vancraeyenest et al., 2020). In the first macaque study applying this approach, double transduction was performed on the propriospinal neurons using the HiRet (Highly efficient Retrograde gene transfer) vector as a retrograde vector, and the adeno-associated virus vector serotype 2 (AAV2) as an anterograde vector to test whether the indirect pathway in the spinal cord through the propriospinal neurons contributes to motor dexterity (Kinoshita et al., 2012). As a result, the reach and grasp movements with the forelimb were impaired 2–5 days after the oral administration of DOX. This dysfunction gradually recovered with the continued administration of DOX. When DOX was administered again after the washout period, the same dysfunction reappeared. These findings indicate that an indirect pathway is involved in motor dexterity.

Ninomiya and colleagues used this technique to block the prefrontal pathway from the ventral premotor cortex (PMv) to the MPFC (Ninomiya et al., 2020). They injected AAV-DJ as an anterograde vector into the PMv and rAAV2.retro as a retrograde vector into the MPFC. They blocked this pathway by continuously administering Dox while the monkeys were performing the task to learn the correct choice from their own and partner’s errors. The results showed that this PMv-MPFC pathway is involved in monitoring the actions of others to make optimal choices.

This technique is relatively minimally invasive since once double transduction is performed, pathway-selective blockade can be achieved by oral administration of DOX. Although the blockade of signal transmission with this method is reversible, it has low time resolution, and takes several days to be effective after the administration of DOX. In addition, long-term administration of DOX can induce the formation of compensatory circuits. Therefore, it is difficult to compare response profiles with and without intervention in the same neuron. This would require combining Tet-on TeNT with recording techniques, such as implantable electrode arrays or calcium imaging, to enable long-term tracking of identical neurons.

### Optogenetics

Manipulation of neural activity using optogenetics in non-human primates began around 2010, and dozens of studies have been reported to date (for review, Galvan et al., 2018; Tremblay et al., 2020). In optogenetics, light-sensitive proteins (opsins) are produced by neurons themselves through genetic engineering to manipulate neuronal activity. The activity of these neurons is then excited or suppressed by light stimulation. Optogenetics allows for more fine-tuned interventions than conventional methods, even when applied locally using specific promoters. For example, some studies have manipulated neural activity in a cell type-specific manner by selectively expressing opsins in dopaminergic, GABAergic, or Konio cells (Klein et al., 2016; Stauffer et al., 2016; De et al., 2020).

Pathway-selective manipulation in macaques using optogenetics is primarily performed by non-selective opsin expression in neurons in a certain brain region with photostimulation of their axon terminals in a particular region projected from those neurons (Inoue et al., 2015; Galvan et al., 2016; Amita et al., 2020; Maeda et al., 2020). As an example of an application to the prefrontal cortex, Inoue and colleagues expressed ChR2 in FEF neurons, delivered photostimulation to their axon terminals in the superior colliculus, and recorded neural activity using optrodes (Inoue et al., 2015). As a result, changes in excitatory or inhibitory activity were observed in the superior colliculus neurons, and eye movements toward the response field corresponding to the stimulation site were elicited. The latency of evoked saccades was slower than that of electrical stimulation, which may be because the effect of optogenetic stimulation of axon terminals is rather moderate compared to electrical stimulation of cell bodies and takes time to cross the threshold to evoke saccades. Recently, Fortuna and colleagues (Fortuna et al., 2020) performed local injections of commonly used optogenetic constructs into the macaque FEF and the dorsal premotor cortex, and anatomically examined long-range axonal projections to parietal and visual areas, providing useful insights for future studies of attentional networks using pathway-selective manipulations.

Optogenetic manipulations have a high temporal resolution on the millisecond time scale, and changes in neural activity are quickly reversed when the light stimulus is terminated. Therefore, it is possible to manipulate and compare neural activity at different times during a behavioral task. Such fine temporal manipulability is a major advantage for dissecting neural circuit function. When using optogenetics, neural activity may be affected by the heat effect of photo-stimulation. Therefore, it is advisable to include control experiments using a viral vector that does not contain opsin sequences. The study mentioned above suggests that changes in the activity of local neurons at the terminal area tend to be relatively mild, with longer latencies and smaller amplitudes than direct electrical microstimulation. Pathway-selective manipulations tend to affect fewer cells than non-selective local manipulations; thus, networks in the prefrontal cortex that are relatively distant from inputs and outputs may be less susceptible to behavioral effects. As discussed below, to induce robust behavioral changes through pathway-selective manipulation in the primate prefrontal network, technological innovations are required to simultaneously stimulate a larger group of neurons. Furthermore, the implantation of light stimulators is highly invasive and poses health risks for their clinical application in humans.

### Chemogenetics

In chemogenetics, the target neurons express artificial receptors that are sensitive only to a specific exogenous ligand that is not normally present in the body. The exogenous ligand is then systemically or locally administered to activate or suppress the activity of the transduced neurons. The most widely employed method in chemogenetics is to express designer receptors exclusively activated by designer drugs (DREADDs), which are modified G-protein coupled receptors (Armbruster et al., 2007; Roth, 2016).

The number of applications of chemogenetics to non-human primates is smaller than that of optogenetics, with only a dozen cases (Eldridge et al., 2016; Grayson et al., 2016; Nagai et al., 2016, 2020; Upright et al., 2018; Raper et al., 2019; Hayashi et al., 2020; Deffains et al., 2021; Hirabayashi et al., 2021; Mimura et al., 2021; Oguchi et al., 2021b; Oyama et al., 2021, 2022; Allen et al., 2021; Jeurissen et al., 2022). These included studies that examined the effects of local interventions on widespread neural networks by fMRI to observe whole-brain network changes, while locally expressing DREADDs and systematically administering the ligand. For example, Grayson and colleagues expressed hM4D_*i*_, an inhibitory DREADD, in the bilateral amygdala of macaque monkeys, then systemically administered the ligand clozapine-N-oxide (CNO) (Grayson et al., 2016). Resting-state functional connectivity MRI revealed that inactivation of the amygdala affected not only monosynaptic pathways connecting it to other brain regions, but also cortico-cortical connections linking regions such as the MPFC, OFC, anterior cingulate, and anterior temporal cortices, causing widespread functional connectivity disruption. They also found the brain regions (the amygdala and temporal pole) that best explained these changes through analysis using communicability in graph theory. Hirabayashi and colleagues expressed hM4D_*i*_ in the hand index finger (D2) region of the unilateral primary somatosensory cortex (SI_*D2*_) and analyzed its effects on grasping behavior and sensory-evoked fMRI signals (Hirabayashi et al., 2021). The administration of deschloroclozapine (DCZ), a highly selective and metabolically stable DREADD ligand (Nagai et al., 2020), causes delays in grasping movements that require precise dexterity with the contralateral hand. An fMRI analysis showed that focal suppression of SI_*D2*_ not only suppressed sensory-evoked BOLD signals in that area, but also affected the downstream grasping movement-related posterior parietal cortex (areas 5 and 7) and the secondary somatosensory area (SII). Interestingly, focal inhibition of SI_*D2*_ enhanced the sensory-evoked BOLD signal to the foot sole and caused hypersensitivity to foot sole stimuli. The results suggest lateral inhibition between areas representing different but related body parts in the somatosensory cortex.

Compared to reports of chemogenetic pathway-selective manipulation in rodents (e.g., Carter et al., 2013; Mahler et al., 2014; Stachniak et al., 2014; Gremel et al., 2016; Vasquez et al., 2019), there have been only two reports in non-human primates, both targeting specific pathways in the prefrontal network (Oguchi et al., 2021b; Oyama et al., 2021). Chemogenetic pathway-selective manipulation can be performed by expressing DREADDs in neurons in a specific region and administering ligands locally to axon terminals in one of their projection sites. Indeed, DREADDs are expressed not only in the cell body, but also in axon terminals, and the local administration of these ligands modulates signal transmission at synapses (Stachniak et al., 2014). Oyama and colleagues tested the functional dissection of the pathways from the DLPFC to the dorsal striatum (CdN) and to the lateral mediodorsal thalamus (MD) in macaque monkeys through local DREADD expression in the DLPFC and microinjection of DCZ in these subcortical areas (Oyama et al., 2021). This study used a spatial delayed-response task in which choices were made manually. The food was placed in one of the left or right food wells, and the monkey memorized its position. The food wells were hidden by a screen for a certain period (0.5, 10, or 30 s), after which the monkey chose either the left or right well, and was rewarded if it chose the correct well. When DCZ was administered bilaterally, the error rate in trials with long delay was increased compared to the control condition only when the DLPFC-MD pathway was suppressed, indicating that the DLPFC-MD pathway is involved in working memory. In contrast, unilateral administration of DCZ increased the error rates to the choice of the contralateral side only when the DLPFC-CdN pathway was suppressed. In an additional task with food in both the left and right food wells, suppression of the unilateral DLPFC-CdN pathway increased the probability of choice for ipsilateral food wells, indicating that the DLPFC-CdN pathway is involved in choice preference. This seminal study succeeded in disentangling the functions of two different prefrontal network pathways in the same animal.

**FIGURE 1:**
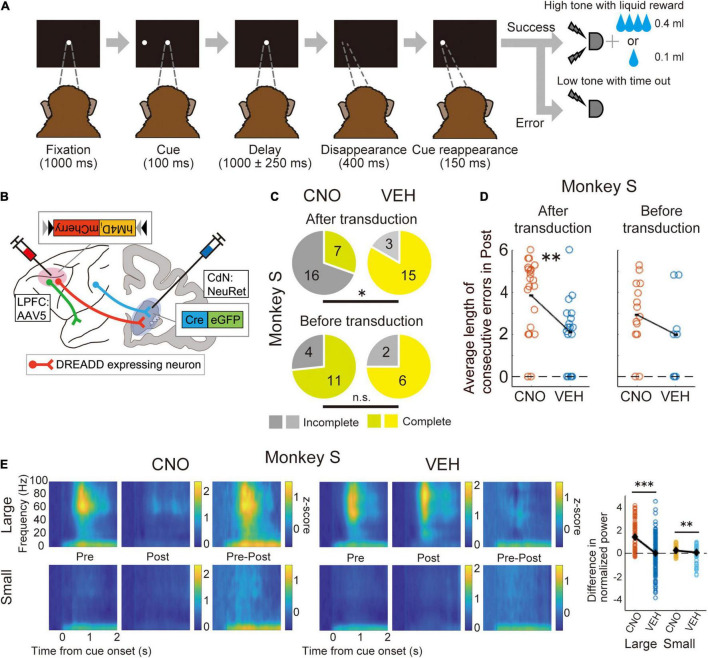
Chemogenetic manipulation of the LPFC-CdN pathway with double-viral vector transduction. **(A)** Time sequence of the one-direction reward (1DR) memory-guided saccade task. The monkeys had to memorize whether the cue was presented to the left or right, and then made a saccade in that direction after the delay period. The allocation of large and small rewards to the left and right changed randomly block by block. **(B)** Illustration of the chemogenetic double transduction. Only doubly transduced neurons whose cell bodies were in the LPFC and axon terminals in the CdN expressed DREADDs. **(C)** Proportion of complete and incomplete sessions before (top) and after (down) double transduction compared between CNO and vehicle (VEH) conditions in one monkey. Yellowish colors indicate complete sessions, and grayish colors indicate incomplete sessions. *P < 0.05, ^**^P < 0.01, ^***^P < 0.001, n.s., non-significant. **(D)** Average length of consecutive errors during the last 160 trials before the end of sessions. Each dot indicates the average length of consecutive errors per session. **(E)** Spectrograms of LFPs recorded from the LPFC divided into large and small reward trials in the CNO and VEH conditions. Pre: The first 160 trials, Post: The last 160 trials, Pre–Post: their subtraction. Color represents normalized power. Time 0 refers to the cue onset. The most right panel shows comparisons between CNO and VEH conditions of the difference in power (Pre-Post) in the time-frequency domain where the cue response was observed. Each dot refers to the subtracted power from each electrode channel. Black diamonds represent mean values. Reproduced with permission from Oguchi et al. (2021b).

Our group used Cre-dependent chemogenetic double-viral vector transduction in macaque monkeys. In this method, the anterograde vector incorporated the “Cre-On” FLEX double-floxed sequence, in which hM4D_*i*_ was included, and the retrograde vector incorporated Cre-recombinase. Using this method, hM4D_*i*_ was expressed only in doubly transduced neurons. First, to anatomically confirm that the double transduction method works in the macaque prefrontal network, we performed double transduction for the LPFC-FEF and LPFC-CdN pathways, and identified doubly transduced neurons labeled with mCherry in the LPFCs (Oguchi et al., 2015). We then performed double transduction in the bilateral LPFC-CdN pathway, which resulted in DREADD expression over a wide range of the LPFC in macaque monkeys that were trained to perform a behavioral task (Oguchi et al., 2021b). The task used here was an oculomotor delayed response task with asymmetric rewards (one-direction reward memory-guided saccade task; 1DR), in which the allocation of large and small rewards to the left and right was randomly switched block-by-block (Figure 1A). The NeuRet (Neuron-specific Retrograde gene transfer) vector, which provides highly efficient retrograde gene transfer, was injected into the CdN to express Cre in neurons with axon terminals there. In the LPFC, AAV5 was used to express hM4D_*i*_ and mCherry in a Cre-dependent manner (Figure 1B). In a recording session, CNO or its control solution (vehicle) was systemically administered to doubly transduced monkeys, and behavior and neural activity were analyzed before and after administration. Neural activity was recorded in the LPFC and CdN using 16-channel U-probe electrodes. CNO administration increased the percentage of sessions in which the monkeys were unable to complete the required number of trials (Figure 1C). CNO administration also increased the average length of the consecutive errors (Figure 1D). Analysis of saccadic eye movements performed to obtain rewards confirmed an increase in impulsivity, with a faster peak saccade velocity and shorter saccade latency. In contrast, we did not observe an increase in saccades in the direction opposite to the correct one, as expected from the working memory deficit. These results suggest that suppression of the LPFC-CdN pathway attenuates inhibitory control functions, such as performing the task to the end with patience and controlling impulsivity in the face of a reward. LFP analysis showed that the positive component after cue onset and the negative component during saccades were attenuated after CNO administration (Figure 1E).

Chemogenetics does not require the implantation of a stimulator in the brain, as in optogenetics, and once the viral vector is injected at the target regions, neural activity can be controlled by systemic administration of the ligand. In the case of chemogenetic pathway selective manipulation by a single transduction, cannula insertion is needed at each session for local administration of the ligand. In contrast, in the case of double transduction, systemic administration is sufficient to achieve pathway selectivity, thus reducing the invasiveness of daily operations. It should be noted, however, that in the case of double transduction, the other regions connected by collaterals of the doubly transduced neurons can also be affected. In animals with large brains, such as macaque monkeys, the potential for inducing behavioral changes is increased if double transduction is conducted over a wide range of target sites, with many relevant projection neurons expressing DREADDs. This is especially true in the neural pathways where anatomical connections are more diffuse. One disadvantage of chemogenetics, however, compared to optogenetics, is that it has an inferior time resolution. Although there are variations depending on the ligand used, with chemogenetics, the effect appears 10 min to 1 h after ligand administration, and lasts for several hours. Therefore, although neural activity cannot be manipulated at a specific time in a behavioral task, the behavior and neural activity before and after ligand administration can be compared within a session. The effect of chemogenetic manipulation is washed out the next day, allowing the experiment to be conducted consecutively under different conditions.

## Strategies for Efficient Gene Expression in the Macaque Brain

One of the difficulties in conducting genetic modification in the brains of non-human primates is that genetic tools that have worked in rodents do not necessarily work well in primates (or their specific genera or species). Therefore, to facilitate pathway-selective manipulation, a viral strategy capable of efficient gene transfer in the brains of each target primate is required.

Adeno-associated virus (AAV) is one of the most commonly used tools for gene transfer in the central nervous system. AAV has several serotypes, each with different infection efficiencies and cell tropisms in the primate brain (Dodiya et al., 2010; Markakis et al., 2010; Watakabe et al., 2015). Among them, AAV1 has a high infection efficiency but low neuronal selectivity, and infects glial cells. Conversely, AAV2 has a high neuronal selectivity and low infection efficiency. Recently, a novel AAV serotype was developed with both high transfer efficiency and strong neurotropism in the macaque brain (Kimura et al., 2021). In this study, a mosaic vector, AAV2.1, was created from the AAV1 and AAV2 capsid proteins that combined their advantages. AAV2.1 has been demonstrated to achieve highly efficient and long-term expression of DREADDs in the macaque striatum and GCaMP in the primary and secondary visual cortices. Our group also used AAV2.1, incorporating the mCaMKllα promoter and GCaMP6s, and successfully expressed GCaMP6s at a high frequency in macaque V1 for microendoscopic calcium imaging (Oguchi et al., 2021a). This AAV2.1 is one of the most promising vectors for use in anterograde infection in the macaque brain.

Alternatively, the Tet-off system has also been used in non-human primates to amplify transgene expression, leading to successful calcium imaging in marmosets and macaque monkeys (Sadakane et al., 2015; Kondo et al., 2018; Bollimunta et al., 2021). In this method, tTA under the control of the Thy1S promoter and GCaMP6f under the control of the TRE promoter were incorporated into separate AAV vectors, and these vectors were injected together. Transgene expression was suppressed by DOX administration and initiated when DOX was withdrawn. In addition to amplifying the expression level, one of the advantages of this Tet-off system is that the transgene expression level can be adjusted by controlling the Dox administration. This advantage is effective in imaging, where appropriate fluorescent signal intensity is required, but may also be useful in optogenetics and chemogenetics when the prevention of cytotoxicity due to overexpression is required.

Double transduction requires an effective retrograde vector in addition to an anterograde vector. AAV6, 8, and 9 have been shown to undergo retrograde transduction in the brains of non-human primates (Masamizu et al., 2011; San Sebastian et al., 2013; Oguchi et al., 2015). For more efficient retrograde transduction, lentivirus-based HiRet and NeuRet vectors have been developed. These vectors are pseudotyped with fusion glycoproteins composed of rabies virus glycoproteins and vesicular stomatitis virus glycoprotein segments (Kato et al., 2011a,b, 2014, 2019; Hirano et al., 2013; Otsuka et al., 2021). The HiRet vector is particularly suited for retrograde transduction from the axon terminals of motor neurons on muscle fibers, whereas the NeuRet vector is suited for retrograde transduction between the subcortical regions. HiRet is less neuron-selective and causes inflammation characterized by microglial and lymphocytic infiltration at the injection site, whereas the NeuRet vector is more neuron-selective and less likely to cause an inflammatory response (Tanabe et al., 2019; Otsuka et al., 2021). Recently, rAAV2.retro, which has been proven to infect retrogradely in the rodent brains, has been tested in macaque monkeys. Weiss and colleagues injected rAAV2.retro and, for comparison, also injected AAV2 into the macaque striatum (Weiss et al., 2020). In the cases with rAAV2.retro, they found a number of retrogradely transduced cells in many cortical and subcortical areas through known afferent projections, whereas in the cases with AAV2, transduced cells were confined within the striatum. Cushnie and colleagues investigated retrograde transduction by injecting rAAV2.retro into multiple areas including the striatum, superior colliculus, FEF, and so on (Cushnie et al., 2020). The results showed retrogradely transduced cells in many areas, but not in some areas with anatomically identified projection pathways, such as the substantia nigra. As noted above, Ninomiya and colleagues successfully used rAAV2.retro in the PMv-MPFC pathway for pathway-selective brocade by Tet-on TeNT (Ninomiya et al., 2020). These studies suggest that rAAV2.retro is a viable option for the cortical-subcortical pathways in macaques, but transduction efficiency will vary from pathway to pathway. Thus, it is important to check beforehand that the retrograde vector to be used works well with the target pathway.

## Strategies for Efficient Light and Ligand Delivery in the Macaque Brain

Macaque monkeys have larger brains than other animal species in which invasive brain research has been conducted, and effective gene transfer and stimulus delivery strategies are required to induce behavioral changes through pathway-selective manipulation. In the prefrontal network, which is relatively distant from both sensory input and motor output, effective behavioral modulation would not be possible without technical efforts, such as expressing artificial receptors in a wide range of neurons and enhancing the delivery of light and ligands to artificial receptors.

One method to achieve widespread transduction is to perform multiple-track injections of viral vectors over a wide area of the target region. One approach is to open the dura mater under anesthesia and inject multiple sites in a single operation (Oyama et al., 2021). Another approach is to have the monkey sit on a monkey chair and then perform the injection process over multiple days with the dura mater left (Oguchi et al., 2021b). Fredericks and colleagues developed a multi-needle injection system that allows the simultaneous injection of multiple sites at precise depths (Fredericks et al., 2020). By injecting manganese ions with the viral vector, they visualized and confirmed the spread of the viral fluid within the target region. With these technical developments, they achieved efficient and uniform transgene expression in the cortical region of macaques. Convection-enhanced delivery (CED), in which fluid is injected under constant pressure, is also used to ensure the wide spread of viral fluids. A single track of vector injection with CED transduced cells over a much larger area than passive spread (Yazdan-Shahmorad et al., 2016). The development and dissemination of such effective injection methods is important for establishing pathway-selective manipulation in macaques as a standard tool. For multiple viral vector injections over a long period (which can occur in macaque experiments), it may be helpful to use viral vectors of different serotypes to prevent humoral immunity from blocking transduction (Mendoza et al., 2017).

In Tet-on TeNT or chemogenetic double transduction, drugs that manipulate neural activity are administered systemically. In this case, the drug is expected to reach a wide area of the brain after crossing the blood-brain barrier. In contrast, pathway-selective manipulation by single-virus vector transduction requires light stimulation or drug microinjection at axon terminals, which must be delivered efficiently to the artificial receptors. In the chemogenetic study using a single transduction described above, researchers used PET to identify *in vivo* the locations where axon terminals of DREADD-positive neurons were densely clustered, and then targeted microinjection of the ligand to these locations (Oyama et al., 2021). Alternatively, CED can be used to facilitate widespread diffusion of the ligand. In optogenetic studies involving non-pathway-selective manipulation, multiple regions of the cortical surface are simultaneously stimulated using optical lasers through a transparent artificial dura mater (Yazdan-Shahmorad et al., 2016, 2018). In these studies, LFPs were simultaneously recorded using microelectrocorticograms (μECoG) with improved transparency for compatibility with optical stimulation. An implantable subdural LED array capable of light stimulation over a wide range of cortical areas has also been developed and used successfully to manipulate neural activity and confirm behavioral changes in the macaque visual cortex (Rajalingham et al., 2021), whereas the delivery of light stimuli to deep cortical layers remains a challenge. These innovations may be particularly useful for less densely connected neural pathways, such as dopaminergic projections from the midbrain to the PFC (Ott and Nieder, 2019).

## Discussion

Over the past decade, pathway-selective manipulation using genetic engineering technologies has been applied to the macaque brain to elucidate brain functions. Thus, some studies have paved the way for fine-grained functional elucidation of the prefrontal network.

Another important challenge in pathway-selective manipulation is recording changes in neural activity from the target pathway or its connected brain regions. As we have discussed, dozens of studies have combined neural activity manipulation with fMRI scans and confirmed that even local manipulation can induce widespread neural activity changes. Although fMRI is useful for examining broad changes in neural activity at the whole-brain level, invasive methods are suitable for analyzing more detailed changes at the intervention site and its connected areas. Multi-contact electrodes, such as U/S-Probes and μECoG, have been used to record spikes and/or LFPs in conjunction with neural activity manipulation (Yazdan-Shahmorad et al., 2016, 2018; Hayashi et al., 2020; Oguchi et al., 2021b). In addition, calcium imaging systems that can switch between two fluorescent colors, which have recently been put into practical use (e.g., nVue system from Inscopix), would be promising. For example, by visualizing DREADD-positive cells in red and observing GCaMP signals in green, it is possible to determine whether the observed neurons are DREADD-positive. It is then possible to analyze how DREADDs positive and negative neurons near the intervention site modulate task-related neural activity after ligand administration. Such an approach would provide a powerful method for elucidating neural circuit function at the mesoscopic level.

Psychiatric disorders such as schizophrenia are characterized as “disconnection syndromes” (Friston and Frith, 1995; Friston et al., 2016), which are caused not by dysfunction in a single brain region, but by that across an extensive neural circuit. Such network abnormalities are associated with a variety of genetic variants that increase the risk of schizophrenia, and have attracted attention as key intermediate phenotypes for the diagnosis and treatment of schizophrenia (Cao et al., 2016). Pathway-selective manipulation of neural activity is useful in creating animal models of psychiatric disorders and understanding their pathology. In particular, the creation of animal models using macaque monkeys, which are closely related to humans and have well-developed PFC, is important for advancing translational psychopathology research. In addition, pathway-selective manipulation of neural activity using viral vectors is a promising novel therapeutic approach for the remediation of specific network abnormalities. In the case of optogenetics, the health risk of cerebral intervention is a major barrier to its clinical application in humans, because it requires the implantation of a light-stimulation device in the brain parenchyma or subdural space. The only exception is its application to retinal neurons, because the lens and vitreous of the eye transmit light well. Optogenetics-based therapies targeting retinal ganglion cells have already been demonstrated in macaque monkeys and humans, and have been shown to restore some vision, such as detecting the presence or absence of visual objects (Gauvain et al., 2021; Sahel et al., 2021). As mentioned above, pathway-selective manipulation can be achieved by oral administration in Tet-on TeNT or chemogenetic double transduction, which is expected to maintain invasiveness at a low level. To promote such innovative therapies, it is essential to further develop basic research and technological improvements in pathway-selective manipulation in non-human primates.

Although there are still few applications of pathway-selective manipulations using optogenetics and chemogenetics to the macaque prefrontal network, further technological innovation and dissemination in macaque studies are expected to promote further research practices in the future. For a detailed elucidation of prefrontal functions, it would be useful to manipulate multiple pathways with the same behavioral task (e.g., one-to-many pathways from one departure area to different destination areas, and conversely, many-to-one pathways from different departure areas to one destination area, as well as feedforward and feedback pathways between interconnected areas) and systematically examine the behavioral and neural effects on these different pathways. At the same time, it would be challenging to develop new behavioral tasks that can appropriately decompose multiple facets to elucidate the abstract functions of the PFC in decision-making. If these challenges can be overcome, research methods using pathway-selective manipulations in the macaque PFC will be a powerful tool for bringing breakthroughs in understanding the neural mechanisms of decision-making.

## Author Contributions

MO and MS conceived of the review topic. MO wrote the manuscript. MS revised the manuscript. Both authors contributed to the article and approved the submitted version.

## Conflict of Interest

The authors declare that the research was conducted in the absence of any commercial or financial relationships that could be construed as a potential conflict of interest.

## Publisher’s Note

All claims expressed in this article are solely those of the authors and do not necessarily represent those of their affiliated organizations, or those of the publisher, the editors and the reviewers. Any product that may be evaluated in this article, or claim that may be made by its manufacturer, is not guaranteed or endorsed by the publisher.
